# Supplementary material for: The ChIP-FRiP pipeline quantifies co-binding and reveals how antibody background contributes to cohesin ChIP-seq patterns
Source: bioRxiv. 2026 Feb 27:2026.02.26.708306. Preprint. [Version 1] doi: 10.64898/2026.02.26.708306 (PMC13160089; doi:10.64898/2026.02.26.708306)
Supplement: Supplement 1 [file media-1.docx]

# Supplementary information for:

**The ChIP-FRiP pipeline quantifies co-binding and reveals how antibody background contributes to cohesin ChIP-seq patterns**

Yao Xiao^1^, Erika C. Anderson^2^, Hadi Rahmaninejad^1^, Elphège Nora^2,3,4^, Geoffrey Fudenberg^1^

1. Department of Quantitative and Computational Biology, University of Southern California, Los Angeles, CA, USA

2. Cardiovascular Research Institute, University of California, San Francisco, San Francisco, CA, USA

3. Department of Biochemistry and Biophysics, University of California, San Francisco, San Francisco, CA, USA

4. Chan-Zuckerberg Biohub San Francisco, CA, USA

#
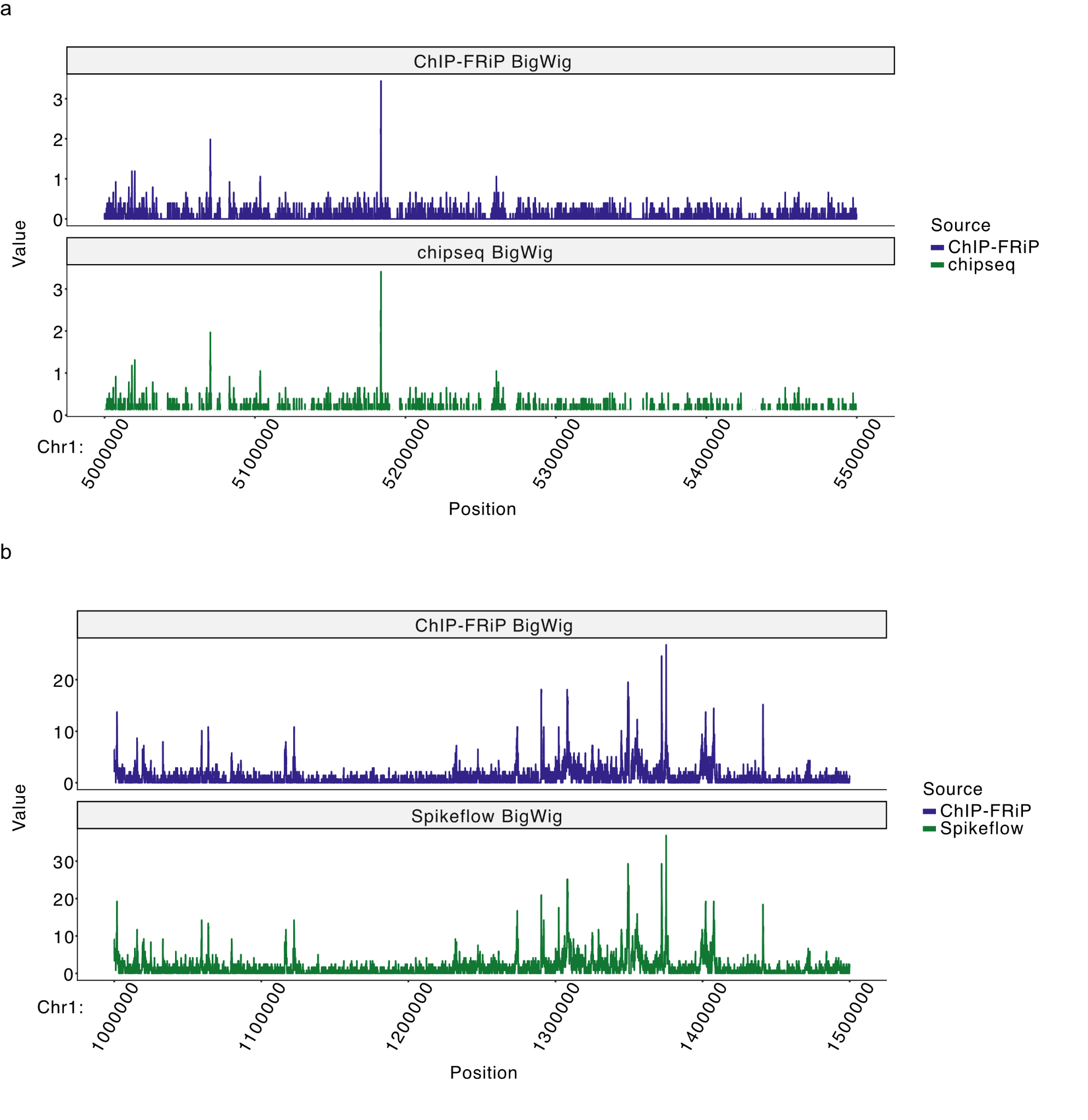


# Fig. S1: ChIP-FRiP yields comparable results to other pipelines for both non-spike-in and spike-in ChIP-seq data. a) Non-spike-in ChIP-seq data from mESCs (SRR5085155[[33]](https://paperpile.com/c/JSDgDs/T4WhR)): ChIP-FRiP is highly concordant with the CPM-normalized bigWig tracks output of the nf-core/chipseq pipeline[[20]](https://paperpile.com/c/JSDgDs/gnLJu) (Pearson’s r = 0.98). b) Spike-in ChIP-seq in hepatocarcinoma cells(SRR20664875[[29]](https://paperpile.com/c/JSDgDs/GgYBF)): ChIP-FRiP is highly concordant with the raw counts output of the Spikeflow pipeline[[19]](https://paperpile.com/c/JSDgDs/h2sMU) (Pearson’s r = 0.96). ChIP-FRiP has the additional option of directly applying the spike-in scaling factor to generate a normalized signal track.

#
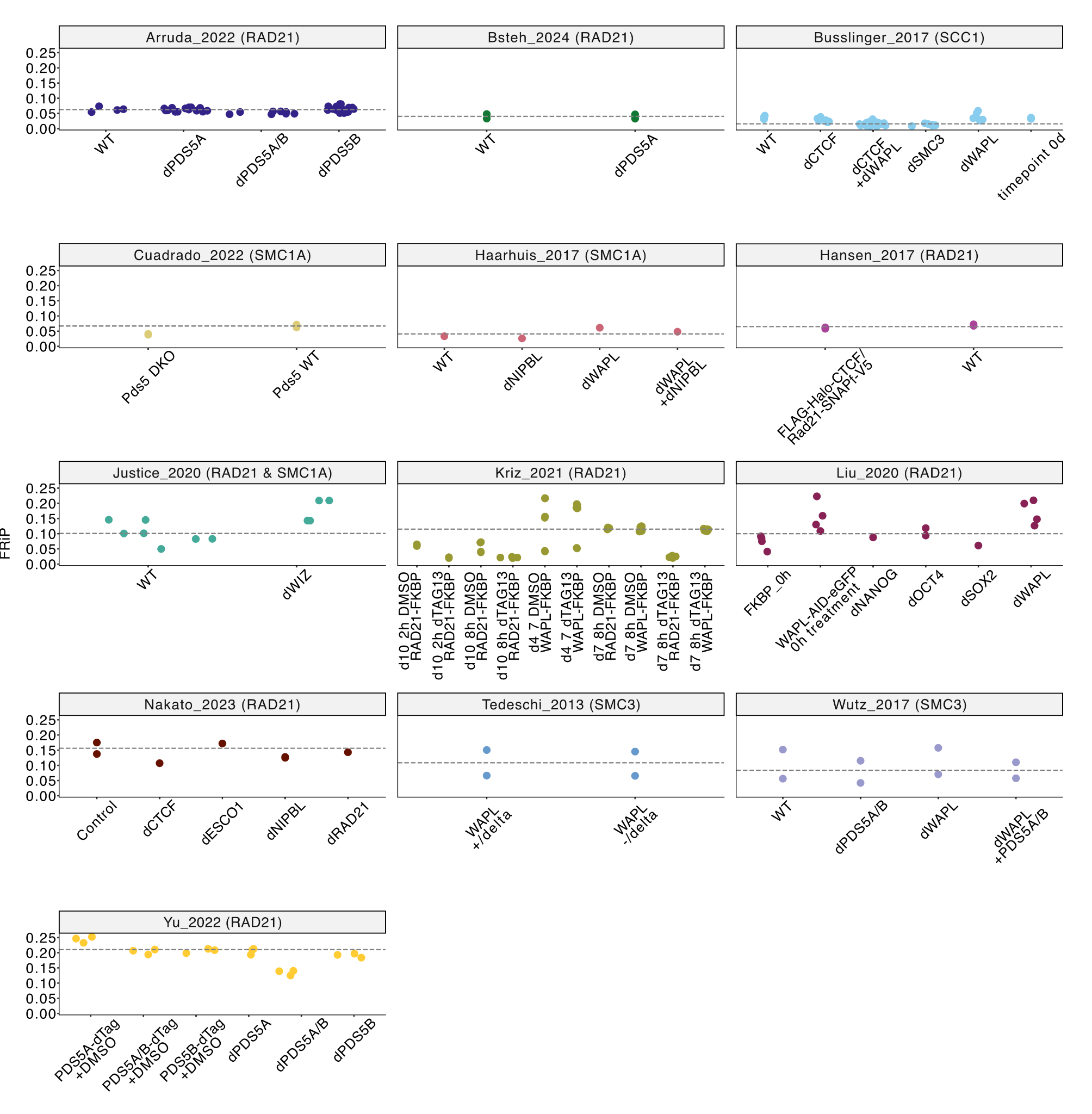
Fig. S2: FRiPs collected from 13 studies reveal a range of behaviors after cofactor perturbation. Each point represents an individual cohesin ChIP-seq sample. Grey dashed lines indicate the median FRiP for unperturbed samples as provided in the metadata of each study.

#
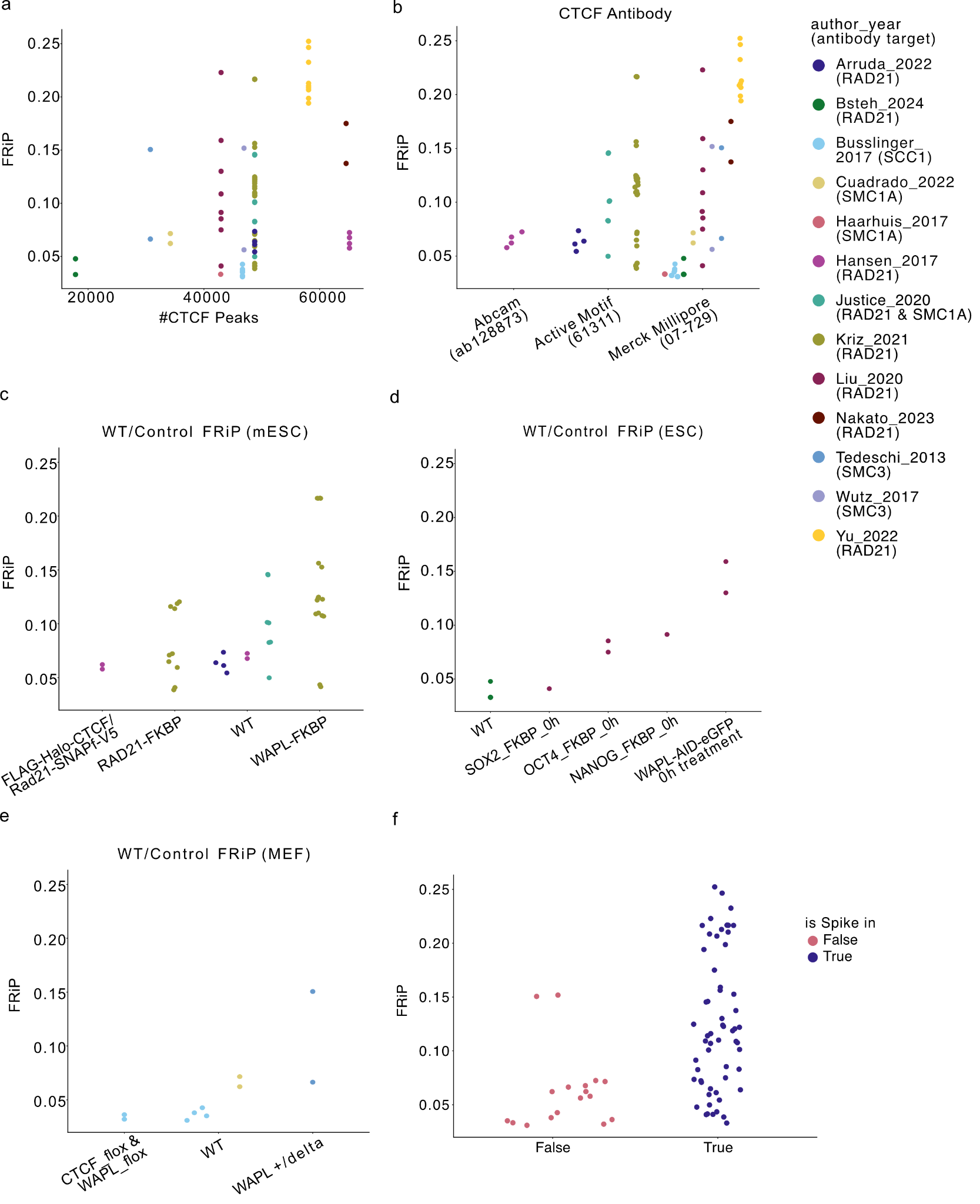


# Fig. S3: Additional covariates that might affect FRiPs in unperturbed datasets. a) FRiPs versus the number of identified CTCF peaks. b) FRiP versus CTCF antibody. c) FRiPs versus TAG type in mESC. d) FRiPs versus TAG type in ESC. e) FRiP versus TAG type in MEF cell. f) FRiP versus spike-in usage.

#
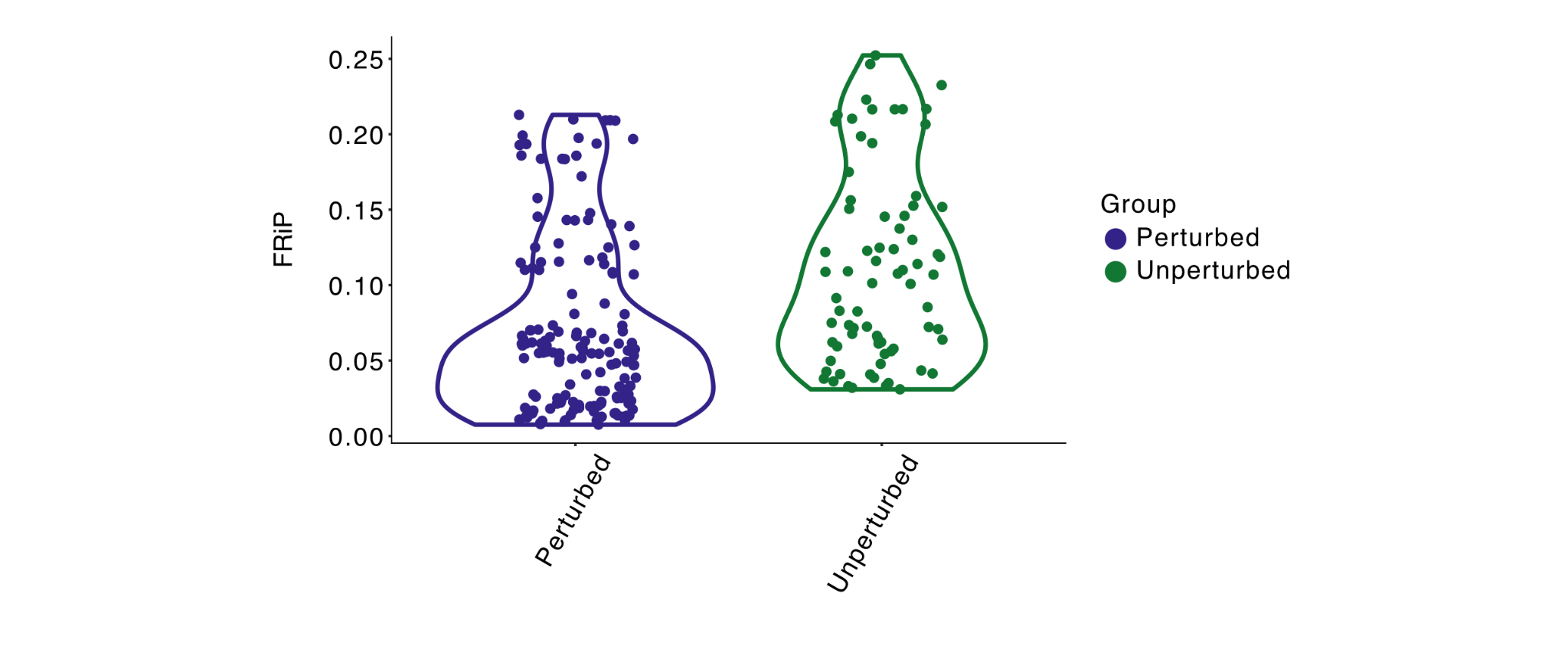


# Fig. S4: Similar FRiP variance between unperturbed and perturbed conditions. Each point represents FRiP computed for either a perturbed or unperturbed sample, aggregated across all studies. The wide dispersion of FRiP across unperturbed samples argues against simple comparisons of FRiPs across studies and conditions without normalization.

#
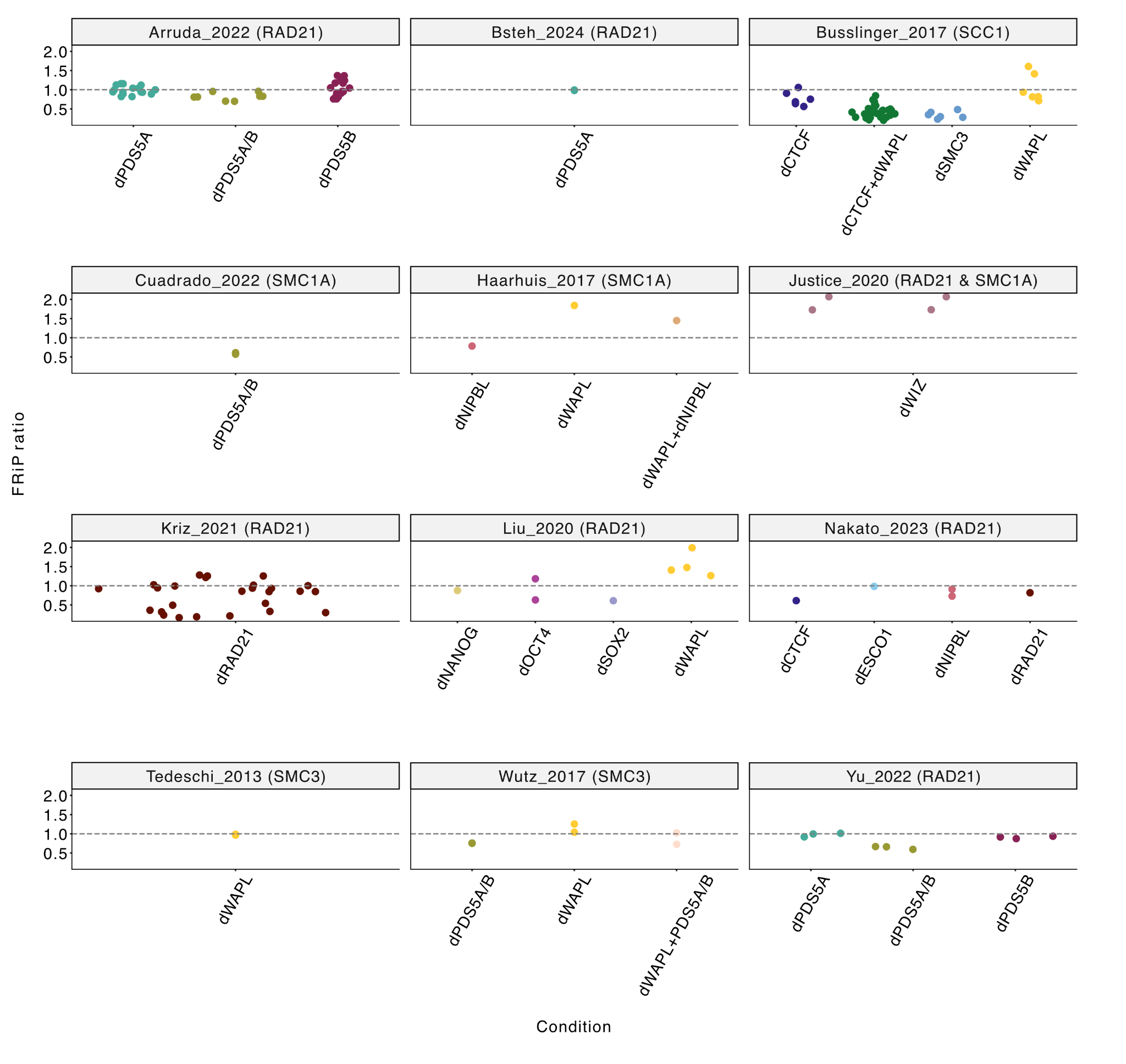
Fig. S5: FRiP ratios across studies. 12 studies have conditions other than unperturbed. Each point represents an individual sample, with colors indicating experimental conditions. Y-axis shows a FRiP ratio of 1.0 indicates no change from unperturbed conditions. The “d” prefix denotes depletion of the corresponding cofactor.

#
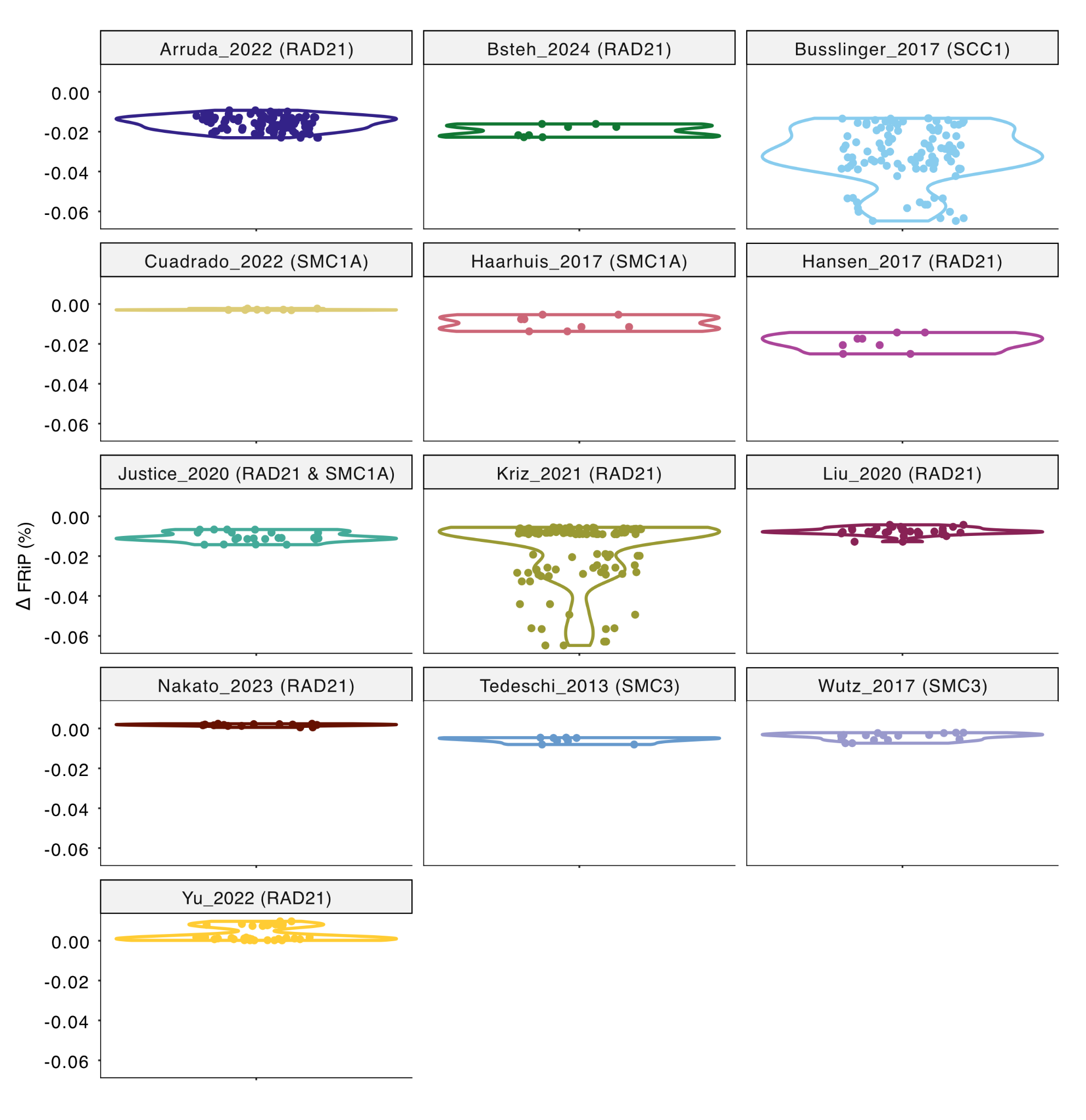


# Fig. S6: Excluding blacklist regions has negligible impact on FRiP. Each datapoint represents the proportional change in FRiP after excluding blacklist regions for each sample from the corresponding study, including both unperturbed and perturbed samples.


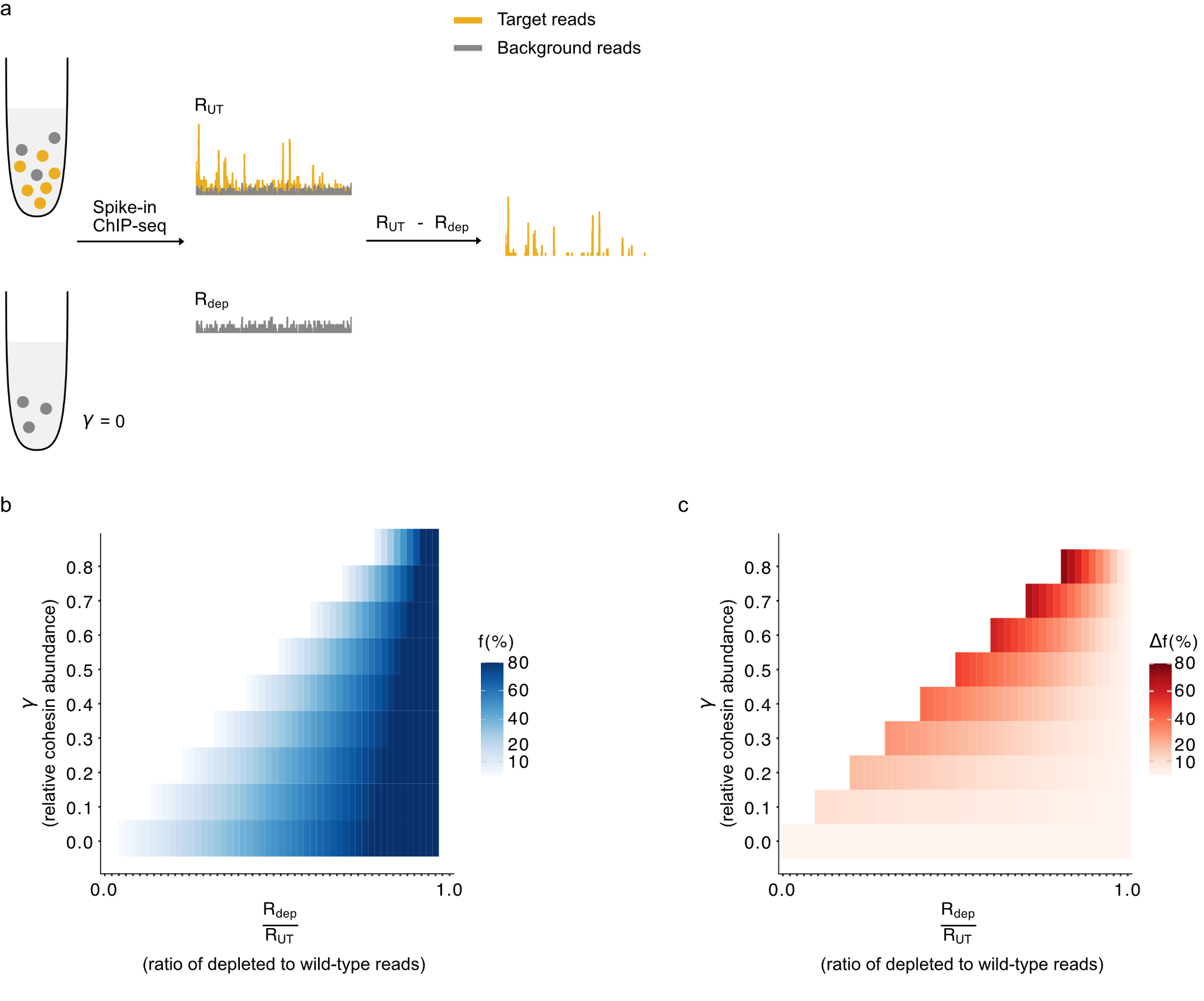


# Fig. S7: Low depletion efficiency with high antibody quality results in large errors in background estimation. a) Illustration of how background can be estimated using spike-in ChIP-seq after depletioning of the ChIPeq protein. R = ChIP-seq reads; UT = unperturbed; dep = depleted; γ = relative cohesin abundance. b) heatmap of background fraction for various ratios of spike-in normalized reads and relative cohesin abundances, illustrating that the same read ratio can correspond to very different background fractions depending on how complete depletion is. c) heatmap of the error in estimated background fraction when complete depletion (γ = 0) is assumed, showing that residual cohesin can substantially bias background estimation.

#
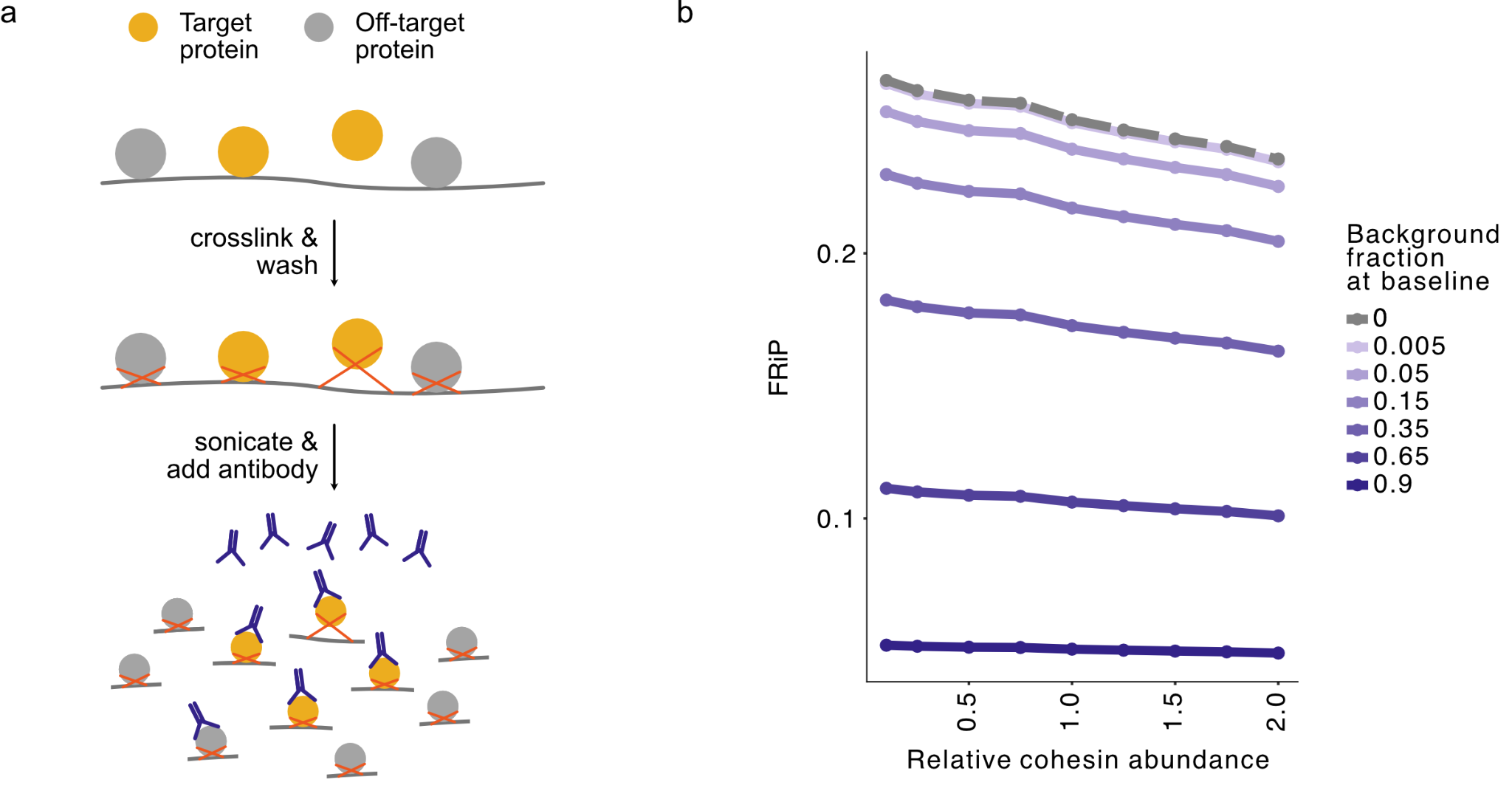


# Fig. S8: Schematic of the alternative proximally-crosslinked ChIP model. a) Formaldehyde randomly crosslinks target proteins proximal to chromatin even when they are not directly bound. With this model, antibodies capture both chromatin-bound and proximally-crosslinked proteins, both of which scale with target protein abundance. b) Under this model, FRiP decreases monotonously as cohesin abundance increases for any background fraction at baseline.

|  | Author,  Year | #Params | Support  Input | Support  no Input | Removes spike-in | Support no spike-in | Alignment software | Filtering software | Peak calling software | Workflow manager |
| --- | --- | --- | --- | --- | --- | --- | --- | --- | --- | --- |
| **ChIP-FRiP** | **Xiao,**  **2025** | **12** | **✓** | **✓** | **✓** | **✓** | **Bowtie2** | **SAMtools** | **MACS2** | **Snakemake** |
| SpikeFlow | Bressan, 2024 | 39 | ✓ | X | ✓ | X | Bowtie2 | SAMtools | MACS2 | Snakemake |
| chipseq | Patel, 2024 | 53 | ✓ | X | X | ✓ | BWA /  Chromap /  Bowtie2 /  STAR | SAMtools & BEDTools & BAMtool & Picard & Pysam | MACS3 | Nextflow |
| ENCODE | Landt, 2012 | 106 | ✓ | ✓ | X | ✓ | Bowtie2 | SAMtools &  Picard | MACS2 | Cromwell |

#

# Table S1: Comparison of ChIP-seq pipelines. Parameter counts shown in the second column were derived from workflow configuration files or provided documentation. X indicates missing functionality for the specified feature. All pipelines mentioned here support both single-end and paired-end data.

#

| **Author_year** | **GEO Accession** | **Species** | **cell type** | **is Spike-in** | **Antibody** |
| --- | --- | --- | --- | --- | --- |
| Arruda_2022 | GSE199356 | Mus musculus | mESCs v6.5 | Yes | RAD21 (Abcam ab992) |
| Bsteh_2024 | GSE194268 | Mus musculus | mESCs | Yes | RAD21 (Abcam ab992); CTCF (Millipore 070729) |
| Busslinger_2017 | GSE76303 | Mus musculus | Mouse embryonic fibroblasts | No | scc1 (abcam ab992; Stag1 (Peters laboratory ID A823); CTCF (Upstate 07-729) |
| Cuadrado_2022 | GSE212151 | Mus musculus | Mouse embryonic fibroblasts | No | SMC1A (Custom made); CTCF (Millipore 07-729) |
| Haarhuis_2017 | GSE95015 | Homo sapiens | Hap1 | No | SMC1A (Bethyl, A300-055a); CTCF (Millipore, 07-72); |
| Hansen_2017 | GSE90994 | Mus musculus | JM8.N4 (mESCs) | No | Rad21 (Abcam ab154769); CTCF (Abcam ab128873) |
| Justice_2020 | GSE137285 | Mus musculus | mESCs v6.5 | Yes | anti-CTCF Active Motif 61311; anti-RAD21 Bethyl A300-080A; anti-SMC1A; Bethyl A300-055A |
| Kriz_2021 | GSE144834 | Mus musculus | mESCs (Tsix-stop) | Yes | RAD21 (Abcam ab992); CTCF (Active Motif 61311) |
| Liu_2020 | GSE135180 | Mus musculus | mESCs & Neural precursor | Yes | CTCF (07-729, Merck Millipore); RAD21 (ab154769, Abcam) |
| Nakato_2023 | GSE196450 | Homo sapiens | RPE | Yes | CTCF (07-729, Merck); Rabbit polyclonal antibody against Rad21; The mouse monoclonal antibody against the acetylated form of SMC3; |
| Tedeschi_2013 | GSE41603 | Mus musculus | Mouse embryonic fibroblasts | No | Smc3 (Bethyl A300-060A); CTCF (Upstate 07-729) |
| Wutz_2017 | GSE102884 | Homo sapiens | HeLa (Kyoto) | No | Anti-CTCF Merck Milipore Cat# 07–729; Anti-SMC3 Peters laboratory Antibody ID:A941; Anti-STAG1 Peters laboratory Antibody ID:A823 |
| Yu_2022 | GSE209849 | Homo sapiens | PLC/PRF-5 (hepatoma) | Yes | ctcf (millipore, 07-729); rad21 (abcam, ab992) |

#

# Table S2: Study metadata. Studies are identified by the first author name and publication year.

# Supplementary note

An alternative model that includes proximally-crosslinked proteins is presented in Fig. S8. In this model, a fraction $\delta$ of $[AC]$ derives from proximally crosslinked cohesins rather than chromatin-bound cohesins. Thus, proximally crosslinked cohesins constitute a second source of background reads, distinct from non-specific antibody binding.

$$Total ChIP reads \propto(1-\delta)*[AC] +\theta+ \delta*[AC]$$

We modified the following equations to account for this updated background composition:

#

#

$f=\frac{\theta+ \delta*[AC]}{[AC] + \theta}$ (Eq. 1)

$\theta=\frac{{(f}_{base} - \delta)}{1 - f_{base}} * [AC]$ (Eq. 2)

$f_{mut}(\gamma) =\frac{\frac{f_{base} * (1 - \delta)}{1 - f_{base}} * [AC]}{[AC] * \gamma+ \frac{f_{base} * (1 - \delta)}{1 - f_{base}} * [AC]} =\frac{f_{base} * (1 - \delta)}{\gamma(1-f_{base}) + f_{base} * (1 - \delta)}$ (Eq. 3)

${R_{dep}} = \frac{f_{UT} * (1 - \delta)}{\gamma(1 - f_{UT}) + f_{UT} * (1 - \delta)}*{R_{dep}}+\gamma*{(1-f}_{UT})*{R_{UT}}$ (Eq. 4)

$f_{UT} = \frac{\frac{{R_{dep}}}{{R_{UT}}} - \gamma}{1 - \gamma- \delta}$ (Eq. 5)

#

If we set $\delta= 0$, then the equations are the same as our primary model.

# 
